# Using entrustable professional activities to guide curriculum development in psychiatry training

**DOI:** 10.1186/1472-6920-11-96

**Published:** 2011-11-23

**Authors:** Philip Boyce, Christine Spratt, Mark Davies, Prue McEvoy

**Affiliations:** 1 Department of Psychiatry, Sydney Medical School-Westmead, Westmead Hospital, PO Box 533, Wentworthville, NSW, 2145, Australia; 2 Northern Melbourne Institute of TAFE, Yarra Bend Road, Fairfield, Vic, 3078, Australia; 3 Royal Australian & New Zealand College of Psychiatrists, 309 LaTrobe Street, Melbourne, Victoria 3000, Australia; 4 Department of Psychological Medicine, Women's and Children's Hospital, 72 King William Street, North Adelaide, South Australia, 5006, Australia

## Abstract

**Background:**

Clinical activities that trainees can be trusted to perform with minimal or no supervision have been labelled as Entrustable Professional Activities (EPAs). We sought to examine what activities could be entrusted to psychiatry trainees in their first year of specialist training.

**Methods:**

We conducted an online survey of Fellows of the Royal Australian and New Zealand College of Psychiatrists (RANZCP).

**Results:**

The majority of respondents considered initiating patients with the common medications, discharging patient suffering from schizophrenia, bipolar disorder or following a crisis admission, conducting risk assessments and managing psychiatric emergencies were activities that trainees could be entrusted with by the end of the first stage of training.

**Conclusions:**

Four activities were identified that trainees should be entrusted with by the end of their first year of training. Each of these activities comprises a set of competencies in each of the CanMEDS roles. When a trainee is unable to satisfactorily perform an EPA, deficits in the underpinning competencies can be a focus for remediation. Further EPAs are being identified in areas of more specialised practice for use within more advanced training.

## Background

Over the past 5-10 years there has been a change in which postgraduate specialist training takes place with a move towards competency based curricula rather than the more traditional time-based apprentice approaches of old [1-3]. The Royal Australian and New Zealand College of Psychiatrists (RANZCP), in line with many specialist colleges, is moving to implement a competency based training program. Its current training program is a time-based curriculum with 3 years of basic training and 2 years of advanced training. A summative assessment (comprising a written assessment, an Observed Clinical Interview and an OSCE) takes place at the end of basic training prior to moving into advanced training.

The move to implement a competency-based curriculum has come about for several reasons. First, in line with contemporary educational thinking, we recognise that the outcome of a training program is to produce specialists that demonstrate that they are competent, across a range of roles, in dealing with contemporary medical problems [4]. In order to ensure that the College's training program fulfils its social obligation in producing such specialists, training has to be aligned with this outcome; this can only be adequately achieved through the use of a competency-based program.

Second, within the RANZCP, we had identified issues of concern within the extant training program. The time to complete training was considerably longer than the five years with the mean time to complete training being 7.4 years. This issue is of particular concern when there are considerable workforce shortages along with increasing demand for specialist psychiatrists. Third, we have had a poor pass rate in our summative assessments suggesting that the training program did not prepare the trainees for these assessments. Fourth, there was considerable trainee dissatisfaction, particularly in the tension between service commitments and time spent in training activities. Finally, we recognised that our training program was not adequately equipping trainees for their future work as consultant psychiatrists, suggesting poor alignment in the training program.

The RANZCP will be implementing a competency-based program from 2013, with some pilot studies being carried out in 2012. In moving towards a competency-based program we elected to adopt the CanMEDS framework [5,6], following an extensive literature review [7] as the most appropriate framework for our purposes. A dedicated committee of the College's Board of Education was established to oversee the development of the new curriculum. The members of this committee had expertise in various aspects of postgraduate education. It included Directors of Training, supervisors and psychiatrists with expertise in training and assessment. Consumers and carers and a trainee representative were also included within this committee. This committee could be considered as an 'expert' panel overseeing the project. As part of this committee's work, we consulted with international educationalists to gauge their opinions regarding the development of our new program.

As part of the process we held extensive consultations with Directors of Training across Australia and New Zealand. Feedback from these groups was provided to the overseeing committee. Whilst these consultations were not formally established as focus groups, the discussions that took place in them were fed back to the committee effectively fulfilling the role of focus group work.

Our new training program will be in three stages; general adult psychiatry for one year to acquire basic competencies; two years spent acquiring competencies in a range of areas of practice (with mandated rotations in Child and Adolescent psychiatry, Consultation and Liaison psychiatry) and finally the acquisition of advanced competencies in a specific area or areas of practice. A purposeful and rigorous program of Workplace-Based Assessments (WBA) - Case-based Discussions, Mini-CEX, Observed Clinical Activities - and summative assessments will support this program.

While workplace-based assessments are recognised as being reasonably valid assessments of competencies, they are generally designed to assess a specific competency. They do not assess across the range of roles expected of a competent psychiatrist. Assessing competencies in this way runs the risk of 'atomising' them rather than bringing them together. This could end up in the situation where a trainee may have a set of competencies (adequately demonstrated in WBAs) but is unable to integrate them across roles that are necessary to demonstrate competent performance.

WBAs do not adequately assess the *actual *clinical duties and responsibilities that trainees have. They are often required to deal with emergency clinical situations at times when a supervisor is not available and there are currently no formalised mechanisms of determining what level of responsibility should be accorded to a trainee at different levels of training.

To overcome this problem we plan to introduce the concept of Entrustable Professional Activities (EPA) to give a more wholistic picture of competence and define what a trainee is entrusted to do with differing levels of supervision.

ten Cate has introduced the concept of EPAs [8,9], as activities, or units of work, that a trainee can be trusted to perform competently. He outlined a set of criteria for these activities: they are essential professional work that can only be carried out by qualified staff, they specify knowledge, skill and attitudes that can be acquired through training, they lead to recognised output of professional labour, can be independently executable within a specific timeframe, they are observable and measurable in process and outcome and lead to a conclusion (done well, or not well). Importantly, they encompass a *set *of competencies across *different roles*.

An EPA will enable a supervisor to know when a trainee can be trusted to carry out specific tasks with minimal or no supervision. They can be mapped to the CanMEDS roles and aligned with the learning objectives of a clinical rotation. Entrustment would reflect not only confidence in a trainee being able to competently complete an activity, but also contribute to the trainee being able to recognise when they need additional supervision

In order to determine what would be appropriate activities that could be entrusted within the first stage of our new training program the curriculum committee elected to survey College Fellows (psychiatrists who have successfully completed their training with the RANZCP) to ascertain their opinions regarding the potential value and use of such activities as EPAs and to identify if the proposed activities could be classed as EPAs for the first year of training, when the trainees are in the earliest developmental stage in their trajectory to acquire Fellowship.

## Methods

All RANZCP Fellows were invited to complete an anonymous online survey which was constructed using SurveyMonkey. The curriculum committee, had previously, through its development work, identified a set of activities that trainees would be expected to carry out in the early stages of training with varying levels of supervision. The committee defined an EPA for the purposes of the survey and included the description in the survey introduction. The identified activities had good 'face validity' as they were determined by expert psychiatrists with extensive experience in psychiatry and postgraduate psychiatry training. Furthermore the face validity was evidenced in that forms of the identified EPAs were already present in the training and assessment program. The survey asked College Fellows to consider whether a trainee should be entrusted with the specific activity by the end of the first stage of training prior to moving on to rotations in specific areas of practice. Fellows were asked to rate whether competence in the proposed areas should be mandatory, very important, somewhat important or of little importance.

The activities that were asked about included initiating various treatments, discharging patients, engaging in family education sessions, conducting risk assessments, admitting patients without supervision, crisis assessments, referring for laboratory investigations, performing neurological examinations and formulating a case.

The survey invited the Fellows to identify other potential EPAs. The survey included an opportunity to collect qualitative data through free text feedback.

The survey was posted on the RANZCP website and an invitation to respond to the survey was sent out in two ways; first, in the College's regular online newsletter (psych-e-bulletin) and then followed up with a group email to all 2736 College Fellows. No further reminders were sent out as in our experience that has not substantially increased the response rate.

## Results

Four hundred and eighty college Fellows (n = 2736) responded to the online survey, a response rate of 17.5%. Table 1 shows the proportion of respondents rating the activities as 'mandatory', suggesting that the activity should be 'entrusted' by the end of the first stage of training.

**Table 1:** Proportion of participants that rated the activity as mandatory prior to progressing to the next stage of training.

Activity	*Number of responses*	Proportion endorsing
*Initiating treatment with*		

Antipsychotic	*476*	**0.82**

Antidepressant	*473*	**0.80**

Mood stabilizer	*473*	**0.75**

CBT	*473*	0.23

Brief psychotherapy	*471*	0.27

Long-term psychotherapy	*473*	0.13

*Discharging a patient from hospital suffering from*		

Schizophrenia	*476*	**0.73**

Bipolar disorder	*476*	**0.72**

An adjustment disorder	*476*	**0.61**

Crisis admission following self-harm	*475*	**0.70**

*Conduct a family education session for*		

Schizophrenia	*476*	0.42

Bipolar disorder	*476*	0.41

Depression	*471*	0.41

Admitting a patient without supervision	*457*	0.56

Conduct a risk assessment	*458*	**0.82**

Carry out crisis assessment and handover report to duty consultant	*456*	**0.77**

Acute assessment and immediate management of psychiatric emergencies	*455*	**0.77**

Perform appropriate lab investigations to exclude medical complications	*459*	**0.69**

Perform a neurological exam	*456*	0.52

Formulate a clinical case	*457*	**0.62**

Non-pharmacological management of a patient with personality disorder	*457*	0.19

Design a biopsychosocial care plan	*457*	0.52

Complete a discharge summary (without supervision)	*457*	**0.63**

Facilitate a follow-up appointment at a community clinic	*457*	0.58

Consent a patient for ECT	*457*	0.51

Perform ECT	*457*	0.31

Acute sedation of a patient	*457*	**0.66**

Initiate a brief substance misuse intervention	*457*	0.29

Non-pharmacological management of an anxiety disorder	*457*	0.36

Pharmacological management of an anxiety disorder	*457*	0.30

**Figure 1:**
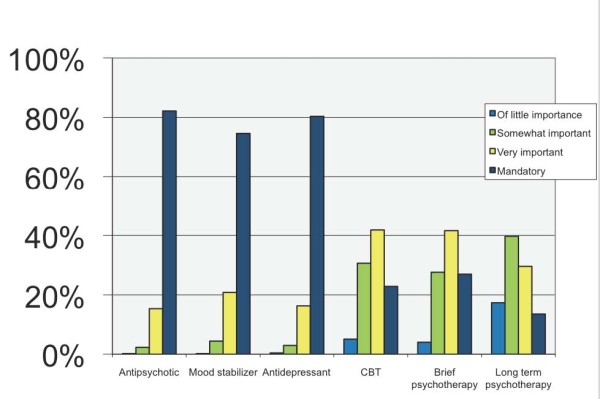
**Responses to question asking whether initiating treatments should be an activity a trainee could be expected to perform by the end of the first stage of training**.

The majority of respondents (over 70%) considered initiating patients with the common medications should be an activity that trainees could be entrusted with by the end of the first stage of training. By contrast, they did not consider initiating a course of cognitive behaviour therapy, brief psychotherapy or long-term psychotherapy as activities that should be entrusted to trainees in this early phase of their training as shown in Figure 1.

There seemed to be broad agreement that trainees could be entrusted to discharge patients suffering from schizophrenia or bipolar disorder from hospital as well as discharging patients following a crisis admission after an episode of self-harm. However, there was lower endorsement for discharging patients suffering from an adjustment disorder.

There was strong agreement that trainees at this stage in their career could be entrusted to conduct a risk assessment, conduct a crisis assessment with handover and report to the duty consultants, and be involved in the acute assessment and immediate management of psychiatric emergencies. The ratings were lower with regard to performing appropriate laboratory investigations to exclude medical complications and in formulating a case or completing a discharge summary.

The majority of respondents did not consider conducting family education sessions for the major psychiatric illnesses, managing a patient with a personality disorder without the use of medication, performing ECT or the nonpharmacological and pharmacological management of anxiety disorders should be entrusted in the first year of training. Some activities were considered mandatory by over 50% (but less than 70%) of respondents, including admitting a patient without supervision, performing a neurological examination, designing a biopsychosocial care plan and consenting patients ECT.

Respondents identified further activities that they considered could be included as EPAs, however many of the activities they identified were specific competencies or areas of knowledge, rather than clinical activities that could be described as 'entrustable' based on our acceptance of ten Cate's (2007) definition.

## Discussion

While online surveys are now widely used in survey research, there remains considerable debate about the best strategies to assure response rates that will allow one to generalise from the data. Historically, low response rates characterise online surveys conducted by our College; given this, we were satisfied that the available data as 'exploratory data' could justifiably inform our deliberations.

In developing a new curriculum for the RANZCP's training program we have elected to use Entrustable Professional Activities as an overarching assessment of competence. These EPAs provide a content focus for WBA, with assessments for each of the EPAs being undertaken early in a rotation; it is intended that these will inform the development of a learning plan, devised by the supervisor and trainee. Before embarking on this we conducted a survey of College Fellows to ascertain their views about activities that could be 'entrusted' to trainees by the end of the first stage of training prior to moving on to train in specific areas of practice. The activities they endorsed are the same activities that trainees often carry out with minimal or no supervision in their day-to-day clinical work. Activities that required more expertise or would not be expected of trainees in this early stage of training did not gain endorsement. This suggests that there is good understanding of EPAs as we presented the concept, particularly the developmental nature of entrustment among College Fellows who responded to our survey, many of whom are engaged in the supervision and teaching of trainees.

Based on the results of this survey and what we considered should be expected of our trainees, the curriculum development group decided on a set of four EPAs that trainees will be required to have assessed during Stage 1 of their training. The survey results were discussed by the curriculum committee at length, especially with regard to how many EPAs should be selected and which one would cover the main areas of competence for this particular stage of training. The committee considered that discussing with a family a diagnosis of schizophrenia was an important EPA as it covered basic knowledge, communication skills, dealt with a disorder commonly seen by our trainees at this stage of their training and is something that trainees are expected to do. The four selected are;

1. completing a discharge summary;

2. initiating antipsychotic medication in a patient with schizophrenia;

3. leading the multidisciplinary team discussion regarding the care of a patient; and

4. carrying out a diagnostic explanation to a family about a young adult's psychiatric illness.

The decision to recommend four EPAs in the first year of training was a pragmatic one based on the survey responses as outlined above. This is a new concept and we wish to conduct more pilot work testing their acceptability, utility and feasibility. Second, we did not want to overburden trainees and supervisors with too many EPAs and their associated WBAs in the initial implementation of this competency-based training program and risk the process being perceived as too burdensome.

Assessment of the EPAs will be by observation of the clinical activity, review of the case notes and discussion with the trainee about the activity. The EPA is a global assessment of competence, as such trainees will be observed in real practice and will either be able to be entrusted (pass) or not entrusted (fail) in the particular activity. For those who are not entrusted it will be necessary to "unpack" the activity (into the relevant CanMEDS roles, as shown in Table 2) to identify the areas of weakness so there can be further remediation supported by specific workplace-based assessment to ensure they have improved that specific skill.

**Table 2:** 'Initiating medication' EPA and the CanMEDS roles.

		Indicative questions
**Medical expert**	The trainee demonstrates the ability to make an accurate diagnosis, has conducted the appropriate assessments, can describe the evidence for the use of the medication, its dosage, interactions and side effects.	*Has an appropriate diagnostic assessment been completed?*
		
		*Is the use of this medication evidence-based?*
		
		*Are there any contraindications to the use of this medication (significant interactions etc)?*
		
		*Is the dosing regime correct?*
		
		*Have appropriate investigations been performed?*
		
		*What plan is there to assess outcome?*

**Communicator**	The trainee shows the ability to explain to the patient the benefits and risks of the medication and how it should be taken and addresses the patients questions.	*Have the reasons for the use of this medication been explained so that the patient is able to understand?*
		
		*Have the benefits of the medication been explained?*
		
		*Have the risks (major side effects) of the medication been explained?*
		
		*Has the patient been explained about what to do should side effects emerge?*
		
		*Has the dosing regime been explained so that the patient understands?*
		
		*Has the need for further investigations been explained?*
		
		*Have the patient's questions been responded to appropriately?*

**Collaborator**	The trainee ensures that members of the MDT (and GP) are aware of how the medication fits in with the management plan	*Has information about the medication been communicated with the MDT?*
		
		*Has information about the medication been communicated with significant others?*
		
		*Has the GP been informed?*

**Professional**	The trainee has obtained informed consent	*Has informed consent been obtained?*
		
		*Are there any conflicts of interest?*

**Scholar**	The trainee is able to apply the evidence from clinical practice guidelines	*What is the evidence base for the medication?*
		
		*What process will be used to evaluate outcome?*
		
		*Can the trainee explain the mechanism of action?*

**Health advocate**	The trainee ensures that the patient is able to access the medication	*Has there been a check on whether the patient can access the medication?*
		
		*Has there been a check on whether the patient can afford the medication?*
		
		*Has the appropriate authority form been used?*

**Manager**	Clear and accurate documentation is completed	*Is the documentation in the case note clear and accurate?*
		
		*Has the medication form (prescription) been completed correctly?*
		
		*Has the use of health resources been considered?*

Initiating medication requires a demonstration of competencies across all the CanMEDS roles. The assessment of the trainee's performance should be conducted in a real life clinical situation under the observation of the supervisor.

Prior to the clinical encounter the trainee should explain to the supervisor the rationale for using this medication, for this patient at this time. This will allow for an assessment of the trainee's knowledge base for the use of this treatment modality.

We are now planning an expanded set of EPAs for specific rotations, such as Child and Adolescent psychiatry, Consultation and Liaison psychiatry, Psychiatry of Old Age, Addiction Psychiatry, Rural Psychiatry and other areas of practice. It is intended that these will become one of the major ways in which competent performance is assessed in the later stages of training.

## Conclusion

The survey data as we have described it gives us confidence that there are clear advantages in using EPAs for our training program. The limitations of the survey are restricted to the low response rate, however as an exploratory study and given the RANZCP's history of low response rates to online surveys, we see the data and our analysis as useful in the broader context of this specific curriculum redevelopment.

**Figure 2:**
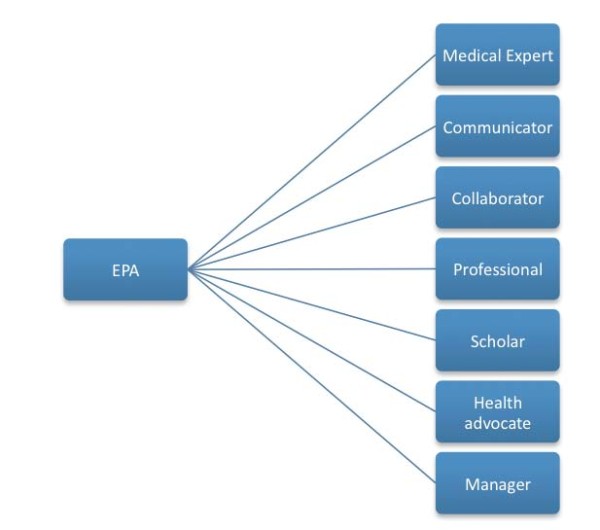
**Relationship between an EPA and the underlying competencies**.

Each EPA comprises a set of underpinning competencies across a range of roles (for our purposes the CanMEDS roles) as shown in Figure 2. This allows the activity to be "unpacked" to identify the role specific competencies as shown in Table 2 for the EPA, initiating an antipsychotic medication to a patient with schizophrenia. This will aid trainees in developing their learning plans so that they have the necessary competencies for the specific EPA. Second, it will guide assessors in what they need to be checking when they come to 'entrust' a trainee for that particular activity (in Table 2 we provide indicative questions that an assessor could think about when assessing a trainee). Third, when a trainee is not performing at a level where they can be entrusted, the supervisor will be better able to guide the trainee in which competencies they need to develop and improve as part of their learning plan. Finally, based on anecdotal feedback received during consultation initiatives as part of the curriculum development, it is anticipated that the use of EPAs will be of great value for clinical directors and supervisors to know what level of responsibility can be accorded to a trainee.

We believe that the use of EPAs in conjunction with a more transparent outcome-oriented training program informed by CanMEDS, will allow us to better prepare trainees and to give supervisors and others engaged in the training program greater confidence in assessing performance. We anticipate that our pilot studies will assist us in developing a more comprehensive understanding of performance based assessment and the complex nature of learning in the clinical setting of postgraduate medical education. Moreover, it offers new opportunities to develop collaborative research across training programs nationally and internationally as medical education continues to investigate the efficacy of competency-based training models and their impact on both learning and patient safety.

## Competing interests

The authors declare that they have no competing interests.

## Authors' contributions

PB developed the conceptual ideas, planned the survey and prepared the manuscript. CS Developed the conceptual ideas, developed the survey and prepared the manuscript. MD constructed the survey, analysed the data and helped prepare the manuscript. PM was a member of the committee developing the new curriculum, reviewed, commented on and edited the manuscript.

All authors read and approved the final manuscript

## Authors' information

PB is Professor of Psychiatry, Sydney Medical School-Western, University of Sydney and formerly, Chair, Curriculum Improvement Project, Royal Australian and New Zealand College of Psychiatrists. CS is Deputy Director Programs, Higher Education, Northern Melbourne Institute of TAFE and formerly educational advisor, Royal Australian and New Zealand College of Psychiatrists. MD is Education Project Officer, Royal Australian & New Zealand College of Psychiatrists. PM is Director of Advanced Training Child and Adolescent Psychiatry, South Australia.

## Pre-publication history

The pre-publication history for this paper can be accessed here:

http://www.biomedcentral.com/1472-6920/11/96/prepub
